# Remote delivery of alcohol and/or substance misuse interventions for adults: A systematic review protocol

**DOI:** 10.1371/journal.pone.0259525

**Published:** 2021-11-02

**Authors:** Neil Howlett, Jaime Garcia-Iglesias, Gavin Breslin, Suzanne Bartington, Julia Jones, Katherine Brown, Wendy Wills

**Affiliations:** 1 Department of Psychology, Sport, and Geography, School of Life and Medical Sciences, University of Hertfordshire, Hatfield, United Kingdom; 2 Bamford Centre for Mental Health and Wellbeing, School of Psychology, Ulster University, Coleraine, United Kingdom; 3 Institute of Applied Health Research, University of Birmingham, Edgbaston, Birmingham, United Kingdom; 4 Centre for Research in Public Health and Community Care (CRIPACC), School of Health and Social Work, University of Hertfordshire, Hatfield, United Kingdom; University of Phayao, THAILAND

## Abstract

**Introduction:**

Alcohol and substance misuse are a public health priority. The World Health Organisation (WHO) estimates that harmful alcohol use accounts for 5.1% of the global burden of disease and that 35.6 million people worldwide are affected by substance misuse. The Coronavirus Disease (COVID-19) caused by severe acute respiratory syndrome coronavirus 2 (SARS-CoV-2) has disrupted delivery of face-to-face alcohol and substance misuse interventions and has forced the development of alternative remote interventions or adaptation to existing ones. Although existing research on remote interventions suggests they might be as effective as face-to-face delivery, there has been a lack of systematic exploration of their content, the experience of service users, and their effectiveness for behavioural outcomes. This review will provide a narrative synthesis of the behaviour change techniques (BCT) contained in interventions for alcohol and/or substance misuse and their association with effectiveness.

**Methods and analysis:**

Systematic searches will be conducted in MEDLINE, Scopus, PsycINFO (ProQuest), and the Cochrane Library. Included studies will be those reporting remote interventions focusing on alcohol and/or substance misuse among adults living in the community and which have a primary behaviour change outcome (i.e., alcohol levels consumed). Data extraction will be conducted by one author and moderated by a second, and risk of bias and behaviour change technique (BCT) coding will be conducted by two authors independently. A narrative synthesis will be undertaken focussing upon the association of BCTs with intervention effectiveness using promise ratios.

**Patient and Public Involvement (PPI):**

The Public Involvement in Research Group (PIRG), part of the NIHR-funded PHIRST, will be involved in refining the review questions, eligibility criteria, data synthesis and dissemination.

**Dissemination:**

Dissemination will be through an academic peer reviewed publication, alongside other outputs to be shared with non-academic policy, professional, and public audiences, including local authorities, service users and community organisations.

## Introduction

Alcohol and substance misuse represent a considerable public health burden worldwide. It is estimated that 5.1% of the Global Burden of Disease is due to harmful alcohol use [1, 2]. Alcohol and substance misuse is also a leading cause of early death among those aged 15 to 49 years [1]. In 2017–2018, 586 780 people or 1.34 per hundred adults had an alcohol dependency in England [3]. Similarly, in the United Kingdom (UK), it is estimated that as many as 314,000 people were using opiates in the years 2016–2017, which would mean an increase of over 13,000 people between 2014–2017 [4]. According to data from the National Drug Treatment Monitoring System (NDTMS), which provides standardized information from most providers of drug and alcohol treatment in England, in 2019–2020, 270,710 adults were in contact with drug and alcohol services [5]. Public Health England estimates that as many as 82% of dependent alcohol drinkers [6] and 52% of opiate and crack-cocaine users [7] are not accessing treatment.

Alcohol and substance misuse pose a significant health service demand. In the UK, there were 7,570 deaths related to alcohol-specific issues in 2019, one of the highest figures since 2001 [8]. Similarly, in 2019/2020, there were 7,030 hospital admissions for substance-related mental health and behavioural disorders (a 21% increase since 2009/2010), 17,000 admissions for poisoning by substance misuse and as many as 99,780 admissions with a primary or secondary diagnosis of substance-related mental and behavioural disorders [9]. More broadly, misuse of illicit substances has been linked to a host of short and long-term impacts on mental health including depression, self-harm, and suicide [10]. This has led to significant national policy efforts to address substance misuse harms, such as the 2017 updating of the Government’s Drug Strategy [11].

Alongside traditional face-to-face interventions for substance and alcohol treatment (such as counselling, brief interventions, and pharmacological treatment), research has also focused on remote delivery of these services in recent years [12]. As more users worldwide gain access to the online technology required to access remote delivery interventions [13], research has evidenced that a multitude of platforms, such as social networking sites [14], may be used to remotely deliver treatments which support a range of substance misuse [15]. While there is limited available primary research data [15], existing literature reviews suggest that both synchronous and asynchronous interventions may be as effective as face-to-face interventions [9, 16] and may be particularly beneficial for groups who are traditionally less likely to access face to face substance and alcohol treatment services, such as women or young people [17].

There are some existing systematic reviews on remote interventions for substance misuse and alcohol treatment [18–20]. These reviews have considered interventions delivered either synchronously or asynchronously through a wide range of technologies, including text-messaging, computers, websites, and smartphone applications. However, these reviews did not examine the active components of the interventions in terms of behaviour change techniques (BCTs). The Behaviour Change Technique (BCT) taxonomy version 1 [21] includes 93 techniques that allow researchers and practitioners to describe and synthesise the “active ingredients” of interventions. Exploring this fine grain detail of the content of an intervention is important for breaking down successful approaches so they can be replicated on a wider scale [21]. Previous reviews that have coded BCTs in this area have either focused on highly specific populations such as pregnant participants [22, 23] or elective surgery patients [24] in face-to-face delivery or focussed exclusively on Randomised Controlled Trials (RCTs) of remote alcohol interventions [16]. To the best of the authors’ knowledge this is the first review to explore the BCTs used in remotely delivered alcohol and substance misuse interventions in the general population.

The COVID-19 pandemic and the associated emergency public health control measures have required significant changes to be made to the delivery of substance and alcohol services across England since early 2020. This has led, in many cases, to the cessation or reduction of face-to-face interventions and a move to remote delivery. Research has established that these changes may have disproportionate negative effects among already vulnerable communities [25, 26]. On the other hand, the changing context has also provided a natural experiment opportunity to test new models of delivery and content [e.g. 27] across services. This review, therefore, seeks to explore the evidence base regarding the BCTs and associated effectiveness of remote delivery of substance misuse and alcohol interventions among adults in community settings.

### Objectives/research questions

This review seeks to answer the following questions:

- What BCTs are contained within remotely delivered alcohol and substance misuse interventions?
- Which BCTs are associated with effectiveness in remotely delivered alcohol and substance misuse interventions?
- What are the experiences of adult service users and providers of remotely delivered alcohol and substance misuse interventions?

### Method

This protocol has been published in the Prospero repository (PROSPERO 2021 CRD42021234116) and follows the Preferred Reporting Items for Systematic Reviews and Meta-Analyses Protocol (PRISMA-P) statement (S1 Checklist) [28].

### Eligibility criteria

The Population-Intervention-Comparator-Outcome-Study design (PICOS) criteria will be used to determine primary study inclusion.

#### Participants

For inclusion in this review, participants must be adults (18 years of age or older) accessing remote interventions for alcohol and/or substance misuse support/harm reduction/recovery and recruited through any settings. Participants should be screened for alcohol and/or substance use risk before inclusion and meet these thresholds: for alcohol, a score of 8 or above on AUDIT (3 or above on AUDIT-C) [29–31]. Studies may be considered if the entry threshold is lower, but the mean of the sample exceeds these scores at baseline. Additional measures, such as Heavy Episodic Drinking (HED) will be considered. For cannabis, studies will be included if they report over half of participants with a cannabis use disorder (CUD) [32] or who reported multiple cannabis-related problems and/or cannabis use on at least half the days in the last week/month. Additional measures, such as DUDIT scores above eight, will be considered [33]. Substance misuse will include the use of opiates or other medications when these have not been prescribed and/or are used in excess of the prescribed dosage.

For the qualitative studies exploring experiences of remote interventions, participants can also be the intervention facilitators/providers in client/patient-facing roles.

#### Interventions

To be considered, interventions will focus on alcohol and/or substance misuse support, harm reduction and recovery delivered remotely. ‘Alcohol and/or substance use’ will include alcohol, illicit drugs, non-medical use of prescription medications, performance enhancement drugs, psychoactive drugs, novel psychoactive substances, and other substances that may lead to a substance misuse. This review will not include interventions aimed at tobacco or nicotine-based products. Interventions to be included will be defined as remote, meaning delivered primarily through phone, computers or mobile devices (e.g. laptop, smartphone) and specific to the service user (as opposed to only readily available libraries of content). Interventions may be synchronous or asynchronous. Interventions targeting multiple behaviours/conditions (e.g. mental health conditions) will be included if data pertaining to alcohol and/or substance use behaviour is reported and discussed separately. There will be no limitation to the duration of remote intervention delivery or timing/frequency of deployment.

This review will not consider interventions for which remote elements were used solely as support to face-to-face elements (e.g. online screening and referral forms, online activities to complete after face-to-face sessions); face-to-face interventions temporarily delivered remotely for more than half their length due to the service user’s inability to access face-to-face services (i.e. due to temporary disability); interventions focusing solely on preventing or reducing alcohol and/or substance use in people planning to get pregnant or during pregnancy; or interventions where the remote components are delivered by post. Studies will be excluded where all or some participants are under 18 years-old, are not living freely in the community or are mandated to access interventions (i.e. as part of criminal justice system sentencing) or are ‘concerned others’ (relatives, family members, friends of the service user).

#### Comparator groups

We expect a wide range of comparators, including, but not limited to, no intervention, service as usual care, face-to-face interventions or hybrid interventions with significant remote and face-to-face content elements.

#### Outcomes

Intervention outcomes will be measured using both self-report and objective methods. Primary outcomes will be those capturing behaviour change related to alcohol and/or substance misuse at a minimum of baseline and post-intervention. There will be no limit to the follow-up duration of outcome measures. Additional secondary outcomes to be considered will be: (i) mental health outcomes (e.g. depression) (ii) physical health outcomes (e.g. associated health conditions) (iii) changes in alcohol and/or substance-use related outcomes (e.g. Accident & Emergency visits) (iv) health inequality related data (e.g. subgroup analysis by Index of multiple deprivation score).

For both primary and additional secondary outcomes, the measures of effect will be differences in means/medians/odds ratio/relative risk between intervention and comparator over time (trials) or differences in means/medians/odds ratio/relative risk between pre and post intervention (single group design), with effect sizes (e.g. Cohen’s d/Hedges’s g) where reported.

#### Study type

Eligible studies will include those described as: qualitative, quantitative or mixed method research design, randomised controlled trials, non-randomised controlled trials/quasi-randomised trials and natural experimental studies (pre- and post- studies, interrupted time series studies).

### Information sources

Searches will be limited to peer reviewed published articles in English language available in: MEDLINE (in process and 1947-date), Scopus, PsycINFO (ProQuest) and Cochrane Library. In addition, the reference lists of each included article will be manually examined for further relevant articles (i.e. cited reference searching). A sample search strategy for PUBMED is attached, which also includes the combination of MeSH terms and keywords used in line with PICO (Table 1). No date or type of publication restrictions will be applied. Filters will be used to only retrieve papers in English language only.

**Table 1:** PUBMED search strategy.

PICO		Themes	Search terms
**Population**	**Alcohol and drug use**	Substance use disorder (MH)	"Substance-Related Disorders"[Mesh]
		Alcoholism (MH)	OR "alcoholism" [Mesh]
		Alcohol abuse*	OR "alcohol abus*"[tiab]
		Substance dependen*	OR "Substance dependen*"[tiab]
		Substance misuse*	OR "substance misus*"[tiab]
		Substance abuse*	OR "substance abus*"[tiab]
			OR"drug abus*"[tiab]
			**AND**
	**Adults**	Adult (MH)	"Adult"[Mesh]
			**AND**
**Intervention**	**Intervention**	Digital Intervention*	"Program Evaluation"[Mesh]
		Digital program*	OR "digital intervention*"[tiab]
		Online intervention	OR "digital program*"[tiab]
		Online program	OR "online intervention*"[tiab]
			OR "online program*"[tiab]
			**OR**
	**Harm reduction**	Harm reduction (MH)	"Harm Reduction"[Mesh]
			**OR**
	**Online remote**	Internet (MH)	"Internet"[Mesh]
		Digit*	OR "Telemedicine"[Mesh]
		Remote*	OR "Cell phone"[Mesh]
		Web	OR "microcomputers"[Mesh]
		(Smart)phone*	OR "mobile applications"[Mesh]
		Phone	OR "social media"[Mesh]
			OR Digit*[tiab]
			OR Remot*[tiab]
			OR Web[tiab]
			OR "smart phone*"[tiab]
			OR Smartphone*[tiab]
			OR "web based*"[tiab]
			OR "web-based*"[tiab]
			OR mobile*[tiab]
			OR "technology-based*"[tiab]
			OR "Telephone"[Mesh]
			OR "phone"[tiab]
			**AND**
**Outcome**	**Behavioural changes**	Reduction of alcohol/substance use	Reduc*[tiab]
			**AND**
**Other criteria**	**Language**	English	"English"[Language]

### Data management/selection process

The results of literature searches will be imported into a citation reference manager (Mendeley), and duplicates removed. One author will screen the title and abstract of all records for inclusion or exclusion. A second author will screen a random sample of 10% of articles. Disagreements will be included for assessment in the subsequent stage. After the title-and-abstract screening, the full-text version of all included studies will be obtained and reviewed independently by two authors, with a third author being consulted to adjudicate any disagreements. A record will be kept of reasons for inclusion and exclusion, as well as disagreements. The selection process will be graphically illustrated using a PRISMA flow diagram [34].

### Data extraction

Two authors will independently extract data from 10% of the included studies into a Microsoft Excel spreadsheet, A third author will moderate agreement on these studies. Given the likely variability between studies included in the review, in terms of design, population, intervention, comparator, outcomes and data type, the data extraction process will be piloted with a sample of the studies included at the full-text-screening stage and then modified if required. One author will then extract the relevant data from each of the remaining studies.

### Data items

Extracted data will include, but not be limited to:

- General study information: author(s), full reference, study title, journal, funding source, declaration of interests, type of study/design, country and location of study, study dates, duration, follow-ups, and sample sizes.
- Descriptive information about the population: sample demographics (subpopulation/group characteristics, gender, ethnicity, age), method of recruitment, inclusion criteria (types of substances, use assessment criteria, assessment of severity, thresholds), groups/arms, clusters, baseline assessments (types of substances, assessment criteria, assessment of severity), additional health concern, other information, sample sizes (at baseline, at completion, at follow-up).
- Information about the intervention: target behaviour, theoretical basis of the intervention, number of sessions or contacts, providers, reported characteristics of the intervention (platforms/tools used, content of the intervention, synchronicity, length, type of remote delivery e.g. website, text message, combination with face-to-face), BCTs based on the BCT Taxonomy version 1 [21].
- Evaluation information: outcome measures (measure of target behaviour, at baseline, at the end of intervention, and at follow-up, where available), effect sizes, service user/facilitator perceptions, accessibility measures, feasibility and usability assessments, drop-out and other performance indicators as reported.

### Outcomes and prioritisation

The primary outcomes are changes in alcohol and/or substance misuse from baseline. This could be measured by self-report such as the quantity of alcohol and/or substances consumed per day/week, measured in alcohol units, number of days of use, number of days since last use, number of substances used, or changes to AUDIT and DUDIT scores. The review will not include interventions that only capture outcomes of behaviour change (e.g. the measures covered in the secondary outcomes). Secondary outcomes to be considered will be: (i) mental health outcomes, including changes in depression, stress, anxiety, dependence, and quality of life; (ii) physical health outcomes, including (perceived) severity of withdrawal and associated health conditions and (iii) changes in alcohol and/or substance-use related behaviours or outcomes, including Accident & Emergency department visits, overdoses, etc. (iv) health inequality related data.

### Risk of bias in individual studies

Due to the diverse types of studies considered, a number of Risk of Bias Assessment Tools will be employed: the Cochrane Risk of Bias tool will be used for randomized controlled trials [35], Risk of Bias in Non-Randomized Studies (ROBINS-I) for non-randomized studies [36], and Critical Skills Appraisal Framework (CASP) for qualitative studies [37]. All studies included in the final selection will be assessed for bias and study quality will be included as a category in the reported table. For trials where a parallel control group is used, it is accepted that random allocation and the blinding of participants and outcome assessor may not be always possible, due to the nature of the interventions and settings. For critical appraisal and risk of bias, two authors will independently assess each study with a third author resolving discrepancies.

### BCT coding

Two authors will independently code BCTs using the BCT Taxonomy version 1 [21] for all included studies and any related paper (e.g. published protocols). The first 10% will also be coded independently by an third author, highly experienced with BCT coding, to check consistency and moderate the initial coding. This process will be repeated again with another 10% of studies halfway through to ensure coding has remained consistent. Inter-rater reliability between the two primary coders will be assessed using the reliability coefficient, Krippendorf’s α [38].

### Data synthesis

Due to the expected heterogeneous nature of the studies, this review will include a narrative analysis of the findings following the Cochrane Handbook and Synthesis Without Meta-Analysis (SWiM) [39]. The main focus of this synthesis will be the assessment of promise ratios for each BCT that appears in multiple intervention studies. Promise ratios will be calculated using the method established by Gardner et al. [40]. This process involves calculating the number of (very or quite) promising interventions featuring the BCT divided by the number of non-promising interventions featuring the BCT. An intervention will be considered very promising when there is a statistically significant improvement in a primary outcome (alcohol or substance misuse) within the experimental group and the difference is significantly greater than the control group improvement (e.g. an interaction effect). Interventions will be classified as quite promising if there is a statistically significant improvement in a primary outcome (alcohol or substance misuse) within the experimental group or the difference is significantly greater than the control group improvement. Interventions showing no statistically significant difference on either will be considered non-promising. Promise ratios will be calculated separately for alcohol and substance misuse interventions.

The data synthesis will also include a discussion of the types of remote delivery (e.g. website only, direct contact with therapist, synchronicity) and degree of personalization (e.g. personalized normative feedback, participant or intervention-directed goal setting, interactions with individual participants) that occurs in these studies. The relevance of the findings to inform policy decisions around face-to-face, remote, or hybrid interventions delivery during and after COVID-19 will be explored.

### Public and Patient Involvement (PPI)

The Public Involvement in Research group (PIRg), part of the NIHR-funded Central PHIRST will be involved in refining the review questions, eligibility criteria, data synthesis and the dissemination strategy. They will be consulted after data extraction is completed to generate insights that help with data analysis and synthesis. They will be actively involved in the dissemination strategy, both for academic and non-academic audiences. The Guidance for Reporting Involvement of Patients and the Public, Version 2 (GRIPP2) reporting checklist [41] will be used to ensure good quality PPI reporting.

## Discussion

Alcohol and substance misuse pose a significant public health concern, with only a minority of those having a dependency accessing treatment. The COVID-19 pandemic and subsequent changes from traditional face-to-face service delivery to remote delivery have disproportionately affected certain communities, but it has also provided a unique opportunity to test the efficacy of remote interventions. It is of paramount importance to understand the effective components of these interventions so successful approaches can be replicated. To date, there is little research on the influence of the behaviour change techniques utilised and their association with effectiveness in remote substance and alcohol misuse interventions for the general population. In addition, there is a pressing need to capture this evidence given the widescale need for remote delivery in the current COVID-19 context and for the foreseeable future. Therefore, this review fills a gap in the literature providing a detailed analysis for these interventions, alongside the experiences of service users and providers. Finally, this review will be part of a wider evaluation of remote drug and alcohol services during COVID-19.

## Supporting information

S1 Checklist PRISMA-P 2015 checklist. (DOCX)
